# Metabolic changes enhance necroptosis of type 2 diabetes mellitus mice infected with *Mycobacterium tuberculosis*

**DOI:** 10.1371/journal.ppat.1012148

**Published:** 2024-05-10

**Authors:** Abhinav Vankayalapati, Olamipejo Durojaye, Tanmoy Mukherjee, Padmaja Paidipally, Bismark Owusu-Afriyie, Ramakrishna Vankayalapati, Rajesh Kumar Radhakrishnan

**Affiliations:** Center for Biomedical Research, The University of Texas Health Science Center at Tyler, Tyler, Texas, United States of America; University of Washington, UNITED STATES

## Abstract

Previously, we found that *Mycobacterium tuberculosis* (Mtb) infection in type 2 diabetes mellitus (T2DM) mice enhances inflammatory cytokine production which drives pathological immune responses and mortality. In the current study, using a T2DM *Mtb* infection mice model, we determined the mechanisms that make T2DM mice alveolar macrophages (AMs) more inflammatory upon *Mtb* infection. Among various cell death pathways, necroptosis is a major pathway involved in inflammatory cytokine production by T2DM mice AMs. Anti-TNFR1 antibody treatment of *Mtb*-infected AMs from T2DM mice significantly reduced expression of receptor interacting protein kinase 3 (RIPK3) and mixed lineage kinase domain-like (MLKL) (necroptosis markers) and IL-6 production. Metabolic profile comparison of *Mtb*-infected AMs from T2DM mice and *Mtb*-infected AMs of nondiabetic control mice indicated that 2-ketohexanoic acid and deoxyadenosine monophosphate were significantly abundant, and acetylcholine and pyridoxine (Vitamin B6) were significantly less abundant in T2DM mice AMs infected with *Mtb*. 2-Ketohexanoic acid enhanced expression of TNFR1, RIPK3, MLKL and inflammatory cytokine production in the lungs of *Mtb*-infected nondiabetic mice. In contrast, pyridoxine inhibited RIPK3, MLKL and enhanced expression of Caspase 3 (apoptosis marker) in the lungs of *Mtb*-infected T2DM mice. Our findings demonstrate that metabolic changes in *Mtb*-infected T2DM mice enhance TNFR1-mediated necroptosis of AMs, which leads to excess inflammation and lung pathology.

## Author summary

Tuberculosis causes increased life-threatening events in people with immunocompromised conditions such as malnutrition, aging, diabetes, smoking and alcohol abuse. Diabetes is an immunometabolic disease and the tuberculosis-diabetes co-morbidity further affects host cell metabolism. Innate immune cells such as alveolar macrophages are primary cells that play an important role in surveillance and clearance of *Mycobacterium tuberculosis* (Mtb) infection. In the current study, we used alveolar macrophages of type 2 diabetes mellitus mice (T2DM) to study the mechanisms of the host susceptibility against *Mtb* infection. We found that the alveolar macrophages of T2DM mice infected with *Mtb* undergo necroptotic (programmed necrosis) mediated cell death. Metabolic comparison of *Mtb*-infected alveolar macrophages of T2DM mice and *Mtb* infected alveolar macrophages of control mice demonstrates that metabolites 2-ketohexanoic acid is highly abundant and pyridoxine (vitamin B6) is less abundant in *Mtb* infected T2DM mice alveolar macrophages. Further studies demonstrate that 2-ketohexanoic acid enhances and pyridoxine reduces necroptosis and inflammatory responses of *Mtb* infected T2DM mice alveolar macrophages. Additional understanding of metabolite mediated inflammatory responses will help to develop better methods to treat T2DM patients with active tuberculosis.

## Introduction

Tuberculosis (TB) kills 1.3 million individuals annually, and it is estimated that approximately one-quarter of the world’s population has latent tuberculosis infection (LTBI) [1,2], and 537 million of the world’s people are confirmed to be diabetic or prediabetic [3], suggesting that it is important to understand immune responses to *Mycobacterium tuberculosis* (Mtb) during diabetes. In diabetic individuals, type 2 diabetes mellitus (T2DM) accounts for approximately 90% [3]. There is limited information available about the immune responses to *Mtb* in T2DM hosts. Previously, we found that the NK-CD11c+ cell interaction in the lungs of T2DM mice infected with *Mtb* leads to pathological immune responses and enhanced mortality [4]. It is important to understand various mechanisms that cause pathological immune responses and mortality of a diabetic host infected with *Mtb*.

Diabetes mellites adversely affects the anti-TB treatment outcomes in TB patients, aggravate the severity of disease and increases mortality compared with non-diabetic individuals [5–7]. Diabetes mellitus also increases the risk of TB by altering host immune cell metabolism and function [8–10]. Insulin resistance in patients with type 2 diabetes leads to the accumulation of metabolites that can nonspecifically activate macrophages. Repeated activation of mature lymphocytes leads to the production of inflammatory mediators [11]. Despite their protective effect in cancer and infectious disease models, published studies suggest that Th1 cells contribute to inflammatory responses in diabetes [12–14]. *Mtb* infection reprograms the metabolism of macrophages by decelerating metabolic flux, which in turn leads to a quiescent energy phenotype [15]. TB-diabetes comorbidity affects whole-body metabolic changes and adversely influences immune cells to contain bacilli [9], and our published results demonstrate that CD11c+ cells are major cell populations that produce inflammatory mediators in T2DM mice infected with *Mtb* [4].

Cellular metabolism is crucial for all living cells to metabolize nutrients for their energy source. Metabolic checkpoints regulate cellular functions, and metabolic perturbations can initiate both apoptosis and necrosis [16]. Apoptosis is a regulated and beneficial cell death mechanism exerted by host macrophages to eliminate intracellular bacteria such as *Mtb* [17]. In contrast, necroptosis is a regulated form of necrosis induced by various cell death receptors, including tumor necrosis factor receptor-1/2 (TNFR1/TNFR2), interferon receptors, and toll-like receptors [18,19], and it is detrimental to hosts. Tumor necrosis factor-α (TNF-α) acts as a pleiotropic cytokine and stimulates TNFR1-dependent cell survival and death. In *Mtb* infection, TNF-α is known to provide protection against *Mtb* infection, but excessive TNF-α can cause pathology [20–24]. Excessive TNF-α production by macrophages triggers diabetic renal injury in streptozotocin-induced type 1 diabetes mice [25] and endothelial dysfunction [26]. TNF-α induces receptor interacting protein kinase 1/3 (RIPK1/RIPK3)-mediated recruitment of mixed lineage kinase domain-like pseudokinase (MLKL)-dependent programmed necroptosis [27–29]. Necroptotic cell death of *Mtb*-infected macrophages causes enhanced dissemination of extracellular bacilli [22]. In addition, a high *Mtb* burden enhances the necroptosis through the induction of TNF-α secretion [22]. *Mtb* virulence proteins such as ESX-1 (ESAT-6 secretion system-1) and CpnT (channel protein with necrosis-inducing toxin) play a role in activation of RIPK1/RIPK3/MLKL and triggers necroptosis [30]. The role of metabolic alterations and various death pathways in the induction of pathological immune responses in T2DM hosts infected with intracellular pathogens such as *Mtb* is not known.

In the current study, we determined the cell death pathways and metabolic alterations that cause pathological immune responses in *Mtb*-infected T2DM mice. We found that metabolic changes in alveolar macrophages (AMs) enhance necroptosis and excess inflammation in *Mtb*-infected T2DM mice.

## Results

### Necroptosis is the major cellular pathway involved in the cell death of *Mtb*-infected T2DM mouse alveolar macrophages

**Fig 1:**
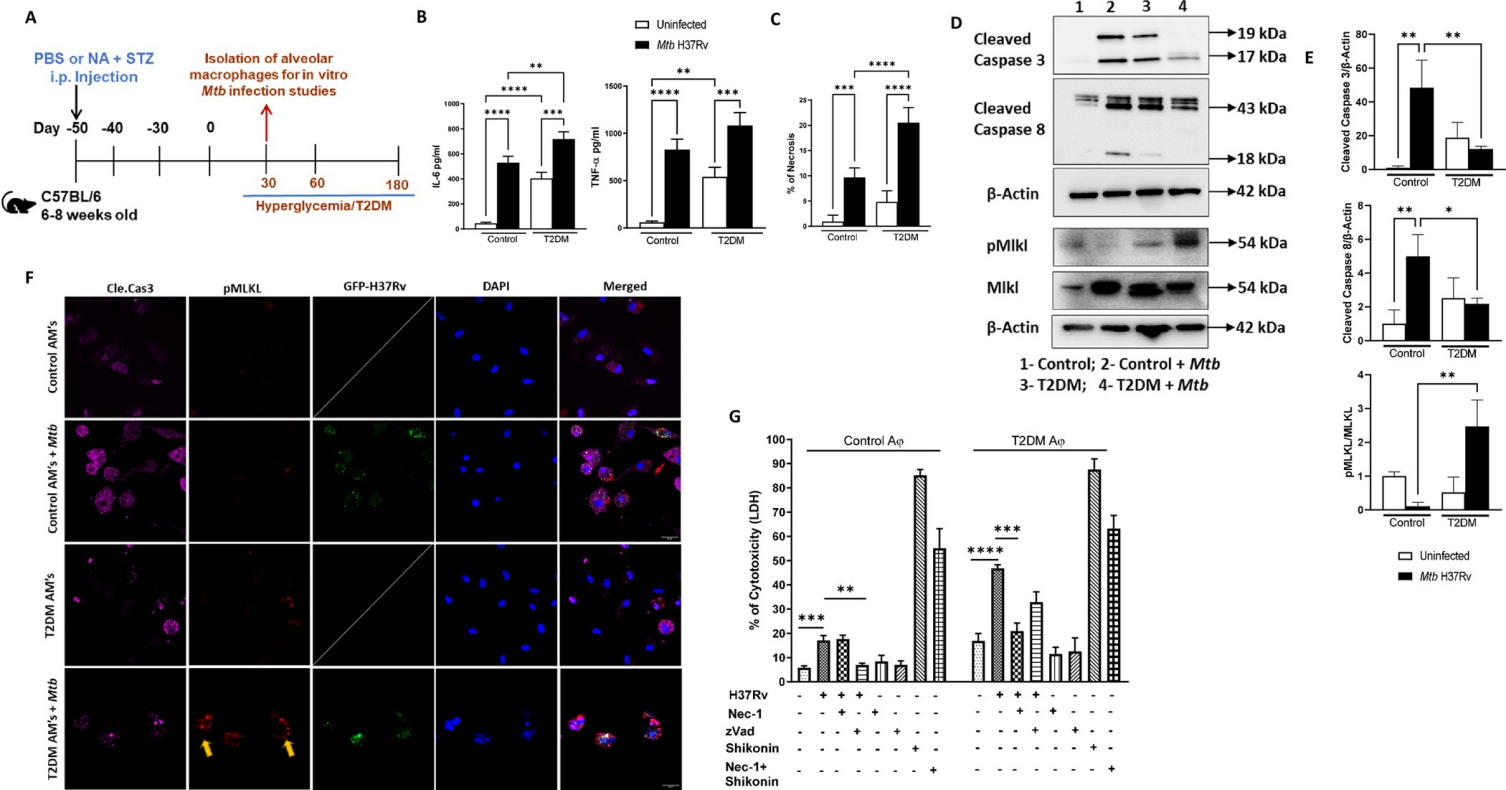
Necroptosis is the major cellular pathway involved in the death of *Mtb*-infected T2DM mouse alveolar macrophages. (A) Schematic representation of the development of T2DM. (B) Alveolar macrophages (AMs) from control and T2DM mice were isolated and infected with *Mtb* H37Rv as described in the methods section. After 24 h, IL-6 and TNF-α levels were measured in culture supernatants by enzyme linked immunosorbent assay (ELISA). (C) Cell death was measured by Annexin/PI staining. (D) After 72 hours, cleaved caspase 3, cleaved caspase 8, MLKL and pMLKL protein expression was determined by western blot and (E) quantification is shown. (F) Cleaved caspase 3 and pMLKL expression was determined by confocal microscopy. Scale bar- 20 μm. Three independent experiments were performed. Each independent experiment was performed using pooled AMs from 3 to 5 mice in each group. The data are shown as the mean ± standard deviation (SD). The statistical analysis was performed by one-way ANOVA followed by Tukey’s multiple comparison test. (G) After 72 hours, lactate dehydrogenase (LDH) release was determined as mentioned in the methods section. Three independent experiments were performed. Each independent experiment was performed using pooled AMs from 3 to 5 mice in each group. The data are shown as the mean ± standard deviation (SD). The statistical analysis was performed by unpaired two tailed t-test. *, p<0.05; **, p<0.01; ***, p<0.001 and ****p<0.0001.

We determined the role of various cell death pathways in the death of *Mtb*-infected T2DM alveolar macrophages. A schematic representation of the development of T2DM is shown in Fig 1A. Alveolar macrophages from control and T2DM mice were isolated and infected with *Mtb* H37Rv as described in the methods section. After 24 hours, as previously found [4], *Mtb*-infected T2DM alveolar macrophages produced significantly higher amounts of IL-6 than *Mtb*-infected control alveolar macrophages (Fig 1B). There was significantly higher cell death in *Mtb*-infected T2DM mouse alveolar macrophages than in *Mtb*-infected control mouse alveolar macrophages as determined by Annexin/propidium iodide (PI) staining (Fig 1C). We also found no significant difference in cell death of uninfected control and T2DM mice alveolar macrophages (Fig 1C). In the above cultured cells, 24 h postinfection, we found the expression of Cas3, Cas8 (apoptosis) and Atg5, Atg7 (autophagy) was significantly downregulated in T2DM mouse alveolar macrophages infected with *Mtb* compared with control *Mtb*-infected alveolar macrophages (S1 Fig). We found no difference in the expression of Cas11 (pyroptosis) and Gpx4 (ferroptosis) between the groups of *Mtb*-infected macrophages (S1 Fig). As shown in S1 Fig, the expression of RIPK3 and MLKL (necroptosis) were significantly increased in *Mtb*-infected T2DM alveolar macrophages compared to *Mtb*-infected control alveolar macrophages.

We confirmed the above findings by Western blotting. *Mtb* H37Rv-infected T2DM mouse macrophages expressed significantly higher levels of necroptotic protein pMLKL than *Mtb*-infected control mouse macrophages (Fig 1D and 1E). In contrast, *Mtb*-infected control macrophages expressed significantly higher levels of cleaved caspase 3 and cleaved caspase 8 (apoptotic proteins) than in *Mtb*-infected T2DM macrophages (Fig 1D and 1E). Confocal microscopy revealed higher expression of membrane-bound (yellow arrow indicated) pMLKL in GFP-*Mtb* H37Rv-infected T2DM mouse alveolar macrophages than in GFP-*Mtb* H37Rv-infected control mouse alveolar macrophages (Fig 1F). As shown in Fig 1G, necrostatin-1 (Nec-1), a necroptosis inhibitor, reduced LDH (Lactate dehydrogenase) release by *Mtb*-infected T2DM mouse alveolar macrophages. zVAD-fmk (N-Benzyloxycarbonyl-Val-Ala-Asp(O-Me) fluoromethyl ketone; pan-caspase inhibitor) significantly reduced LDH release in control mouse alveolar macrophages infected with *Mtb* (Fig 1G). Taken together, these results suggest that T2DM mouse alveolar macrophages infected with *Mtb* undergo necroptosis.

### TNFR1 mediated necroptosis of *Mtb*-infected T2DM mouse alveolar macrophages

**Fig 2:**
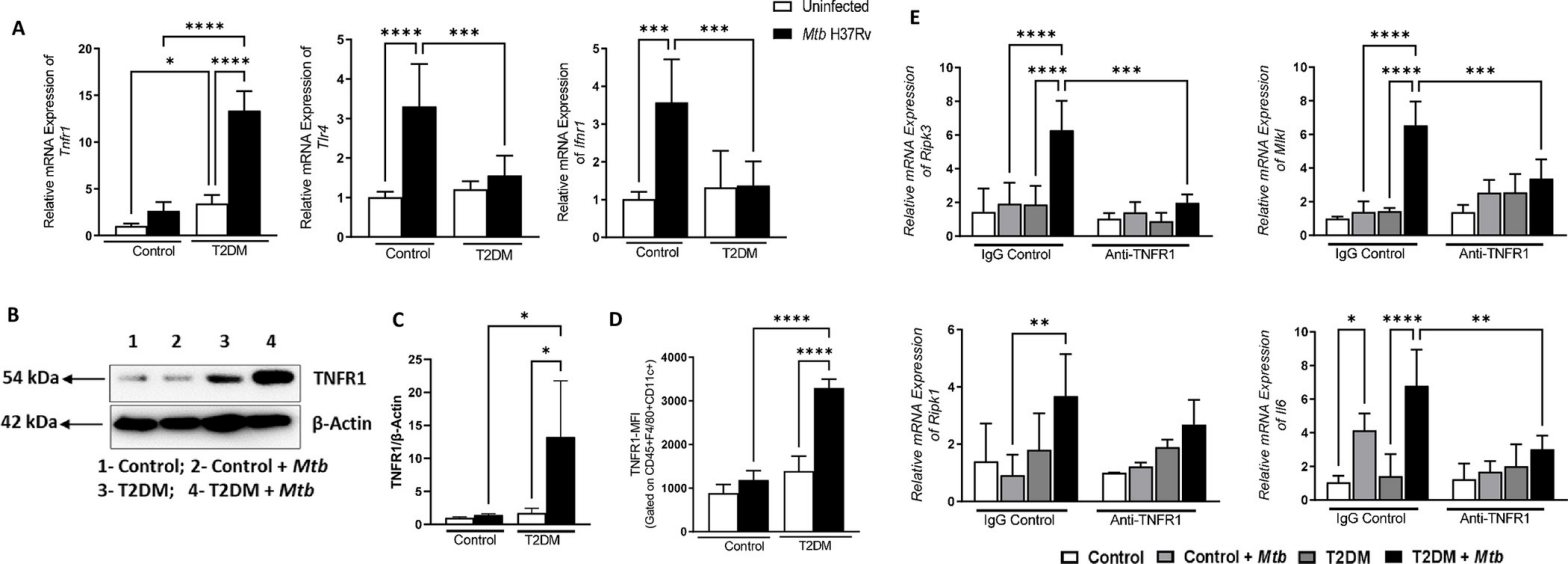
*Mtb*-infected T2DM mouse alveolar macrophages undergo TNFR1-mediated necroptosis. AMs from control and T2DM mice were isolated and infected with H37Rv. After 24 hours, TNFR1, TLR4 and INFR1 expressions were determined by (A) qRT-PCR. After 72 hours of infection with *Mtb*, TNFR1 expression was determined by (B-C) Western blot and quantification and (D) flow cytometry. (E) AMs from control and T2DM mice treated with anti-TNFR1 antibody and infected with *Mtb*. After 24 hours, the mRNA expression of RIPK3, RIPK1, MLKL and IL-6 was determined by qRT-PCR. Three independent experiments were performed. Each independent experiment was performed using pooled AMs from 3 to 5 mice in each group. The data are shown as the mean ± standard deviation (SD). The statistical analysis was performed by one-way ANOVA followed by Tukey’s multiple comparison test. *, p<0.05; **, p<0.01; ***, p<0.001 and ****p<0.0001.

Depending on the disease model, necroptosis is mediated through the activation of tumor necrosis factor receptor 1 (TNFR1), interferon receptor (IFNR) and toll-like receptor 4 (TLR4) pathway [31]. In the above groups of macrophages, TNFR1, IFNR1 and TLR4 expression was determined by qRT-PCR. As shown in Fig 2A, the expression of TNFR1 was significantly increased in *Mtb*-infected T2DM alveolar macrophages compared to *Mtb*-infected non-T2DM alveolar macrophages. In contrast, the expression of IFNR1 and TLR4 is significantly reduced in T2DM mice alveolar macrophages infected with *Mtb* when compared with control mice alveolar macrophages infected with *Mtb* (Fig 2A). TNFR1 expression was confirmed by western blotting and flow cytometry (Fig 2B, 2C and 2D). As shown in Fig 2E, anti-TNFR1 antibody treatment significantly reduced the expression of RIPK3, MLKL and IL-6 compared to isotype control antibody in T2DM mouse alveolar macrophages infected with *Mtb*.

### T2DM mouse alveolar macrophages exhibit differential metabolomic signatures

**Fig 3:**
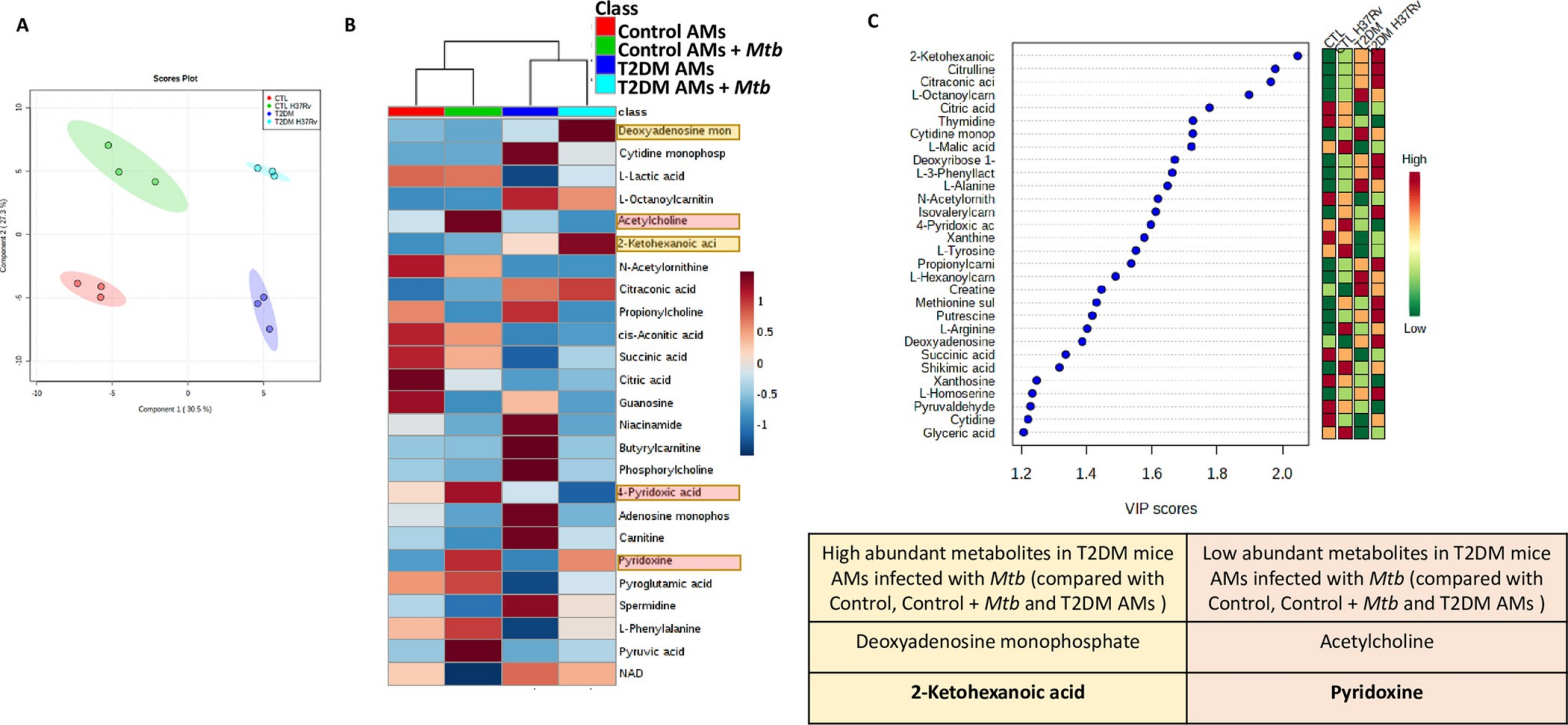
Metabolic profiles of *Mtb* H37Rv-infected control and T2DM mouse alveolar macrophages. AMs from control and T2DM mice were isolated and infected with *Mtb* H37Rv. After 72 hrs, cell lysates were analyzed using LC/MS. (A) Principal component analysis (PCA) plot showing the segregation of samples in the four groups of AMs tested. (B) The heatmap shows the top 25 high abundance and low abundance metabolites. Red indicates upregulated and blue indicates downregulated metabolites in the respective groups. (C) Variable importance of projection (VIP) scores of 25 metabolites (greater than 1.0 based on PLS-DA is shown). On the extreme right, red and green indicate high and low levels of metabolites, respectively (table insert shows selected metabolites). Each independent experiment (n = 3) was performed using pooled AMs from 5 mice in each group.

Metabolic changes can regulate TNFR1-mediated necroptosis [32]. Based on the above findings, we determined whether *Mtb*-infected T2DM mouse alveolar macrophages have differential metabolomic profiles compared to *Mtb*-infected control mouse alveolar macrophages. Alveolar macrophages from control and T2DM mice were isolated and infected with *Mtb* H37Rv. After 72 h, cell lysates were analyzed using liquid chromatography/mass spectrometry (LC/MS). We found a high level of segregation of metabolites in all four groups of mouse alveolar macrophages tested (control, control *Mtb*, T2DM and T2DM *Mtb*) (Fig 3A) by using a partial least squares-discriminant analysis (PLS-DA) algorithm, suggesting a unique metabolomics signature among groups. Among the segregated groups, using the variable importance in the projection (VIP) score, we identified 25 metabolites with VIP scores >1.0 (considered to be the most significant metabolites) in the T2DM mouse alveolar macrophages infected with *Mtb* as the most important in group segregation when compared with all the other groups of macrophages tested (Fig 3B and 3C). We selected the metabolites with the highest VIP scores and heatmap analysis as the most significant in the segregation of metabolic changes (Figs 3B, 3C, and S2A). Of these selected metabolites, deoxyadenosine monophosphate and 2-ketohexanoic acid were highly abundant in *Mtb*-infected T2DM mouse alveolar macrophages, and acetylcholine and pyridoxine (vitamin B6)/4-pyridoxic acid were significantly less abundant when compared with all other three groups of mice alveolar macrophages tested (Fig 3B and 3C). Furthermore, quantitative metabolite enrichment analysis was performed, and the metabolic pathways enriched in T2DM mouse alveolar macrophages infected with *Mtb* compared to control mouse alveolar macrophages infected with *Mtb* are represented (S2B Fig). The results indicated that the phosphotidylcholine biosynthesis, spermidine and spermine biosynthesis and methylhistidine metabolism pathways are highly enriched in *Mtb*-infected T2DM mouse alveolar macrophages (S2B Fig).

### Metabolites alter the expression of TNFR1 by *Mtb*-infected alveolar macrophages

**Fig 4:**
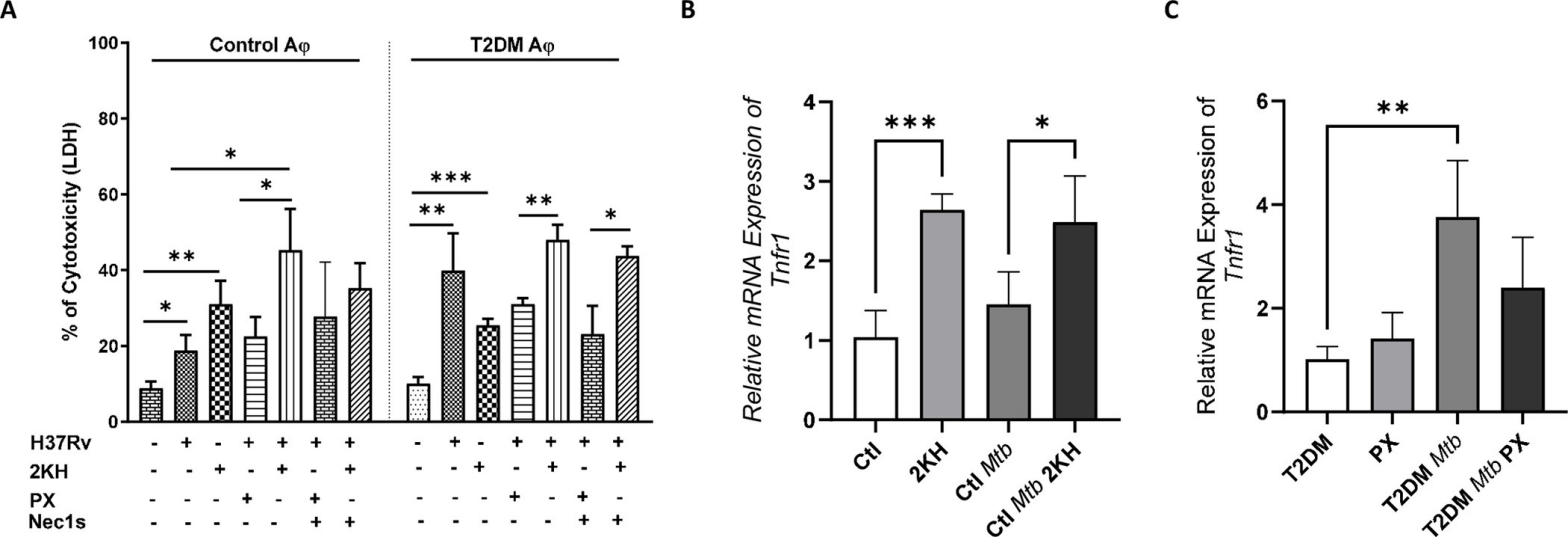
Metabolites alter the expression of TNFR1 by *Mtb*-infected alveolar macrophages. Control and T2DM mouse AMs were infected with *Mtb* H37Rv and treated with 50 μM of 2-ketohexanoic acid (2KH) and pyridoxine (PX) or PBS. (A) After 24h h, LDH release (cytotoxicity assay) was determined. The data are shown as the mean ± standard deviation (SD). The statistical analysis was performed by unpaired two tailed t-test. *, p<0.05; **, p<0.01 and ***, p<0.001. (B-C) TNFR1 expression was determined by qRT-PCR. Each independent experiment was performed using pooled AMs from 3 to 5 mice in each group. The data are shown as the mean ± standard deviation (SD). The statistical analysis was performed by one-way ANOVA followed by Tukey’s multiple comparison test. *, p<0.05; **, p<0.01 and ***, p<0.001.

We determined whether metabolites could alter TNFR1 expression in nondiabetic control and T2DM mouse alveolar macrophages upon *Mtb* infection. First, we tested the cytotoxicity of the metabolites against uninfected control mice alveolar macrophages as mentioned in the methods section (S3A Fig). We found 2-ketohexanoic acid significantly increased cytotoxicity in control mice alveolar macrophages and in contrast pyridoxine reduced the cytotoxicity of T2DM mouse alveolar macrophages infected with *Mtb*, as determined by LDH release assay (Fig 4A). Based on the cytotoxicity, using the optimal concentration (50 μM) of the above metabolites, we next evaluated their effect on TNFR1 expression (as measured by qRT-PCR) by *Mtb*-infected alveolar macrophages. As shown in Fig 4B, 2-ketohexanoic acid increased the expression of TNFR1 by uninfected and *Mtb*-infected macrophages when compared with phosphate-buffered saline (PBS)-treated uninfected and *Mtb*-infected macrophages. Pyridoxine treatment did not reduce TNFR1 expression by T2DM mouse macrophages infected with *Mtb* compared with *Mtb* infected PBS-treated macrophages (Fig 4C). There was no difference in TNFR1 expression in the presence of deoxyadenosine monophosphate and acetylcholine (S3B Fig).

### Metabolite treatment alters TNFR1-mediated necroptosis of *Mtb*-infected alveolar macrophages

**Fig 5:**
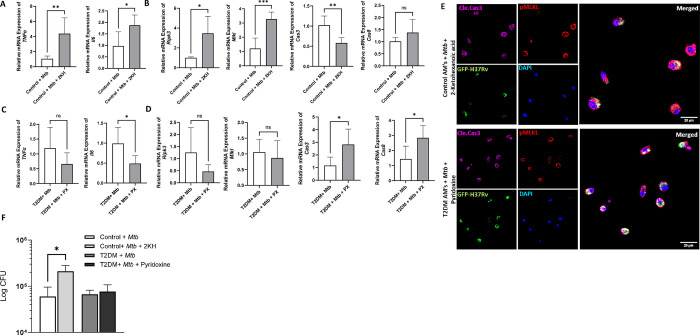
Metabolites alter TNFR1-mediated necroptosis of *Mtb*-infected alveolar macrophages. Control and T2DM mouse AMs were infected with *Mtb* H37Rv and treated with 50 μM of 2-ketohexanoic acid and pyridoxine. After 24 h (A-D), TNF-α, IL-6, RIPK3, MLKL, Cas3 and Cas8 mRNA expression was determined by qRT-PCR. (E) Control and T2DM mouse AMs were infected with GFP-*Mtb* H37Rv (FITC) and treated with 2-ketohexanoic acid (50 μM) and pyridoxine (50 μM), respectively. After 72 h, AMs were immunofluorescently probed with cleaved caspase 3 (far red), pMLKL (red) and DAPI (blue) and images were acquired by confocal microscopy at 63x magnification. Scale bar- 20 μm. (F) Control and T2DM mouse AMs were infected with *Mtb* H37Rv and treated with either 50 μM of 2-ketohexanoic acid or pyridoxine. After 72 hours, the bacterial burden was determined. Data from three independent experiments are shown. Each independent experiment was performed using pooled AMs from 3 to 5 mice in each group. The data are shown as the mean ± standard deviation (SD). The statistical analysis was performed by unpaired two tailed t-test. *, p<0.05; **, p<0.01 and ***, p<0.001.

We determined whether 2-ketohexanoic acid and pyridoxine treatment alter TNFR1-mediated inflammation and necroptosis. We found that 2-ketohexanoic acid significantly increased TNF-α, IL-6, RIPK3 and MLKL mRNA expression and bacterial burden of control mouse alveolar macrophages infected with *Mtb* (Fig 5A, 5B and 5F). In contrast, 2KH significantly reduced the expression of Cas3 and had no effect on Cas8 expression (Fig 5B). In addition, confocal microscopy revealed an increase in membrane-bound pore-forming pMLKL expression in control mouse alveolar macrophages infected with GFP-*Mtb* and treated with 2KH (Fig 5E). Pyridoxine significantly reduced expression of IL-6 and increased expression of cas3 and cas8 by T2DM mouse alveolar macrophages infected with *Mtb* (Fig 5C–5E). Pyridoxine had no effect on TNF-α, RIPK3, MLKL mRNA expression and bacterial burden of T2DM mouse alveolar macrophages infected with *Mtb* (Fig 5C, 5D and 5F).

### Metabolic changes enhance the necroptosis and pathology of *Mtb*-infected T2DM mice

**Fig 6:**
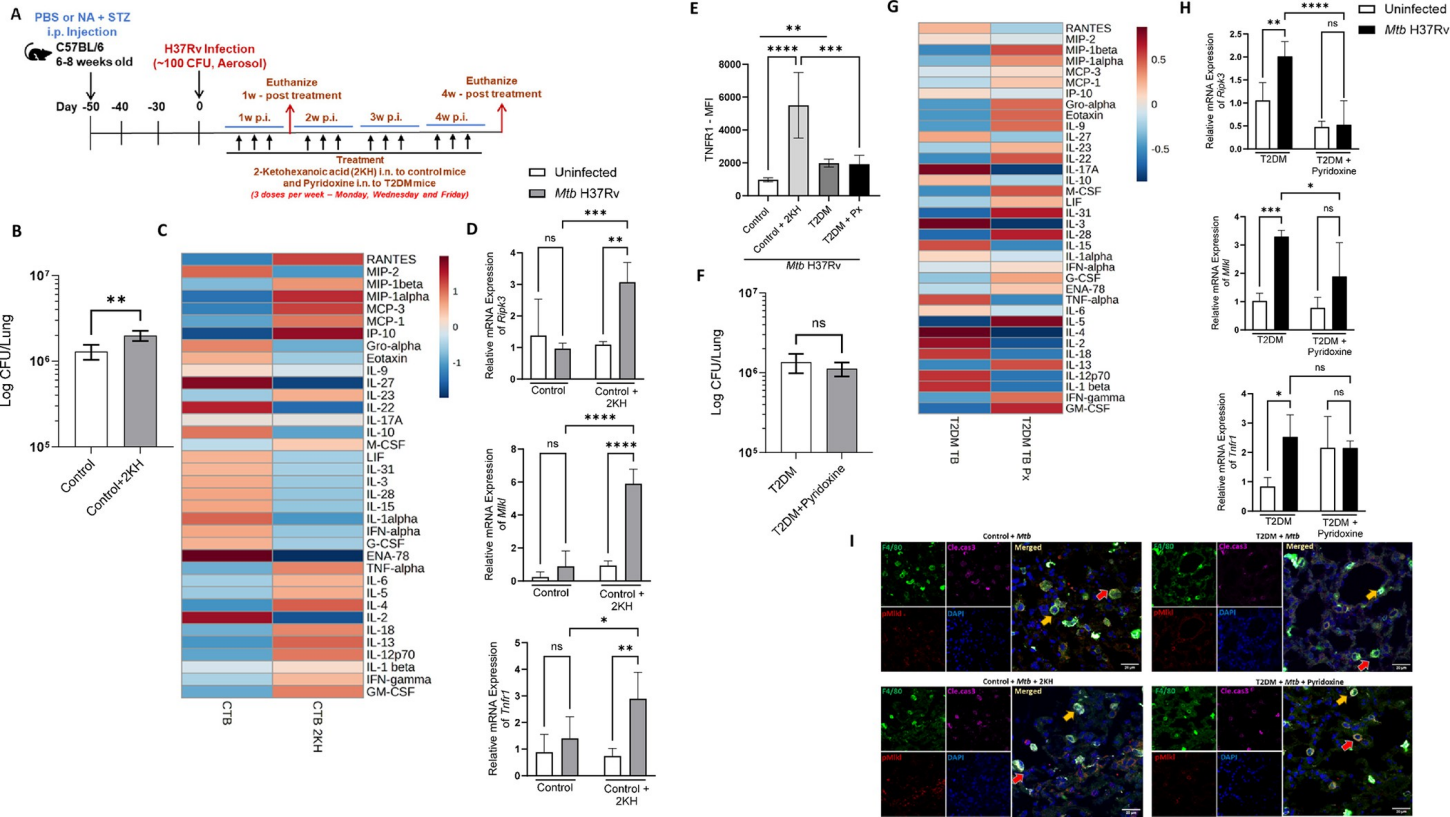
Metabolites treatment alters TNFR1 expression, necroptosis, inflammatory cytokine production and bacterial burden in the lungs of *Mtb*-infected mice. (A) A schematic representation of T2DM induction and intranasal metabolite treatment in C57BL/6 mice is shown. Some of the uninfected and *Mtb*-infected T2DM mice were treated with 2-ketohexanoic acid (20 mg/kg of body weight) or pyridoxine (20 mg/kg of body weight) intranasally as mentioned in mehods. One-month postinfection in 2-ketohexanoic acid-treated mice, (B) Bacterial burden in the lungs was measured. The data are shown as the mean ± standard deviation (SD). The statistical analysis was performed by unpaired t-test. **, p<0.01. (C) Lung homogenates were collected, and cytokines/chemokines were estimated using 36-plex ELISA kit by Luminex and the heatmap data is presented. (D) Expression of necroptotic molecules (RIPK3, MLKL) and TNFR1 was determined in the lungs by qRT-PCR. (E) Flow cytometry analysis was used to measure TNFR1 expression in CD11c+F4/80 cells in 2-ketohexanoic acid- and pyridoxine-treated mouse lungs infected with *Mtb*. Five mice per group were used for each independent experiment. The data are shown as the mean ± SD. The statistical analysis was performed by one-way ANOVA followed by Tukey’s multiple comparison test. *, p<0.05; **, p<0.01; ***, p<0.001 and ****, p<0.0001. (F) Bacterial burden in the lungs of pyridoxine treated *Mtb*-infected T2DM mice was measured. The data are shown as the mean ± standard deviation (SD). The statistical analysis was performed by unpaired t-test. (G) lung homogenates were collected, and cytokines/chemokines were estimated using 36-plex ELISA kit by Luminex and the heatmap data is presented. (H) Expression of necroptotic molecules (RIPK3, MLKL) and TNFR1 was determined in the lungs by qRT-PCR. (I) paraffin-embedded tissue sections were subjected to deparaffinization and immunolabeled with F4/80 (FITC), pMLKL (red), cleaved caspase 3 (far-red) and DAPI. Immunostained sections were imaged under confocal microscopy at 63x magnification. Scale bar- 20 μm. Data are representative of two independent experiments. Five mice per group were used for each independent experiment. The data are shown as the mean ± standard deviation (SD). The statistical analysis was performed by one-way ANOVA followed by Tukey’s multiple comparison test. *, p<0.05; **, p<0.01; ***, p<0.001 and ****, p<0.0001.

We determined the in vivo relevance of the above findings. A schematic representation of the development of T2DM, infection with *Mtb* and treatment of control and T2DM mice with metabolites is shown in Fig 6A. Some of the uninfected control and T2DM mice and *Mtb*-infected control and T2DM mice were treated with metabolites intranasally as mentioned in the methods section (2-ketohexanoic acid was given to control and control mice infected with *Mtb* H37Rv and pyridoxine was given to T2DM and T2DM mice infected with *Mtb* H37Rv).

As shown in S4A Fig, 2-ketohexanoic acid had no effect on bacterial burden, increased CD11c+F4/80+ and CD11c+F4/80+TNFR1+ cells (S4C Fig) and increases levels of IL-1α, IL-1β, IL-22, IL-4, IL-5 and ENA-78 cytokines and chemokine at 1-week post infection compared with *Mtb*-infected control mice (S4D Fig). After 1 month of post infection, in the lungs, 2-ketohexanoic acid moderately but significantly enhanced the bacterial burden (Fig 6B), increased RANTES, MIP-1α, MCP-3, IP-10, TNF-α, IL-6, IL-4, IL-5, IL-13, INF-γ and GM-CSF (Fig 6C) and no difference in CD11b+ and CD11c+ sub-cell frequencies (S6A Fig). We also found 2-ketohexanoic acid enhanced the expression of TNFR1, MLKL and RIPK3 and reduced Cas3 and Cas8 (Figs 6D, 6E and S5A-S5B). Immunofluorescence signals indicated an increased pMLKL-expressing macrophages (Fig 6I, red arrows indicate necroptotic cells and yellow arrows indicate apoptotic cells) and significant increase of lung inflammation determined by hematoxylin and eosin staining in the lung sections of 2-ketohexanoic acid treated *Mtb*-infected mice compared with PBS treated *Mtb*-infected control mice (S6B–S6C Fig).

In pyridoxine treated T2DM mice, one week after infection, there is no difference in bacterial burden (S4B Fig), immune cell populations (S4C Fig) and reduced TNF-α and IL-6 levels (S4D Fig) compared to PBS treated *Mtb*-infected T2DM mice lungs. One month after infection, pyridoxine moderately reduced TNF-α and IL-6 levels (Fig 6G), reduced the number of CD11c+CD11b+ and CD11c+F4/80+ cells (S6A Fig), reduced RIPK3 and MLKL mRNA expression, had no difference in bacterial burden and TNFR1 in lungs of PBS treated *Mtb*-infected T2DM mice (Figs 6E, 6F, 6H and S5B). Immunofluorescence images indicated a reduction in pMLKL-expressing macrophages (Fig 6I; red arrows indicate necroptotic cells and yellow arrows indicate apoptotic cells) and no difference in lung inflammation as determined by hematoxylin and eosin staining (S6B–S6C Fig) in the lung sections of pyridoxine treated *Mtb*-infected T2DM mice compared with PBS treated *Mtb*-infected T2DM mice.

## Discussion

*Mycobacterium tuberculosis* causes tuberculosis and remains a leading cause of death globally [33]. Immunometabolic diseases such as type 2 diabetes exacerbate the risk of tuberculosis [9,34]. Previously, we found that pathological immune responses enhance the mortality of T2DM mice infected with *Mtb* [4]. We also found that macrophages are initial sources of inflammation [4]. In the current study, we investigated the mechanisms that enhance the inflammatory responses of *Mtb*-infected T2DM mouse alveolar macrophages. Among the various cell death pathways that cause inflammation, we found that T2DM mouse alveolar macrophages undergo TNFR1-mediated necroptosis upon *Mtb* infection. Neutralizing TNFR1 reduced expression of necroptosis markers such as RIPK3 and MLKL and IL-6 levels. *Mtb*-infected T2DM mouse alveolar macrophages have a high abundance of metabolites 2-ketohexanoic acid and less pyridoxine (vitamin B6) than *Mtb*-infected non-T2DM mouse alveolar macrophages. In *Mtb*-infected nondiabetic control mice, 2-ketohexanoic acid treatment increased TNFR1-mediated inflammation and necroptotic cell death of alveolar macrophages. In T2DM mice infected with *Mtb*, pyridoxine treatment reduced expression of necroptosis markers RIPK3 and MLKL. Our current study demonstrates that metabolic changes in *Mtb*-infected T2DM mice enhance TNFR1-mediated necroptosis cell death of alveolar macrophages.

Alveolar macrophages are the first cells to respond to *Mtb* infection and play a major role by eliminating intracellular pathogens [35,36]. *Mtb*-infected macrophages undergo either apoptosis or necrosis depending on the multiplicity of infection [37]. Macrophage apoptosis reduces the survival of bacilli and inflammation and enhances antigen presentation [38,39]. In contrast, virulent *Mtb* infection enhances the necrosis of macrophages, which allows the growth of extracellular bacteria and dissemination of disease [40]. In bacterial and viral infections, necrotic cell death is associated with complex death pathways, including necroptosis, pyroptosis, ferroptosis and NETosis [41–45]. We found that alveolar macrophages of *Mtb*-infected T2DM mice underwent necroptotic death (among the different pathways tested, Fig 1C–1E). Under hyperglycemic conditions, alveolar macrophage function, and the expression of various surface receptors is altered [46]. Increased TNF-α and TNF receptor expression is associated with T2DM [47,48]. In the current study, we found that *Mtb* infection enhances TNFR1 expression by alveolar macrophages of T2DM mice (Fig 2A–2D).

TNF-α/TNFR1 induce programmed necrosis (necroptosis) in *Mtb*-infected macrophages mediated through RIPK1, RIPK3 and MLKL [27,45]. Necroptosis is programmed necrosis that differs from other death pathways (apoptosis, autophagy and pyroptosis) in the requirement of a unique signaling pathway associated with the activation of RIPK1, RIPK3 and MLKL [49,50]. There are conflicting reports about the role of necroptosis during *Mtb* infection. Depletion of RIPK3, MLKL and inhibition of RIPK1 had no effect on cell survival, bacterial burden and pathology of *Mtb*-infected macrophages or humanized mice [27,51]. In contrast, activation of RIPK3 and inhibition of caspase-8 induce necroptosis of *Mtb*-infected macrophages [52]. We previously found T2DM causes excess inflammation during *Mtb* infection in mice [4] and in the current study, we found necroptosis of *Mtb*-infected T2DM macrophages contributes to excess inflammation. Necroptosis exacerbates inflammatory responses to infection, which contribute to tissue damage and pathology [53,54]. Bacterial lipoproteins are well characterized to activate the TLR2-mediated apoptotic signaling pathway [55,56]. Innate cells recognize *Mtb* infection by different pattern recognition receptors, including Toll-like receptors (TLRs). TLR1, TLR2, TLR4 and TLR9 play important roles in the activation of the immune response against TB pathogenesis [57]. In addition, type 1 interferon signaling also mediates macrophage necroptotic cell death upon *Mtb* infection [43,58]. Our current study demonstrates that increased expression of TNFR1 by *Mtb*-infected alveolar macrophages of T2DM mice activates RIPK3 and MLKL mediated necroptosis. Anti-TNFR1 antibody treatment of *Mtb*-infected T2DM mice alveolar macrophages reduced inflammatory IL-6 production and expression of RIPK3 and MLKL (Fig 2E). Anti-TNFα neutralization in mice reactivates *Mtb* growth and may worsen the TB disease [59]. In the current study, instead of neutralizing TNF-α, we determine whether T2DM mediated excess inflammation in *Mtb*-infected mice can be regulated by metabolites.

Insulin resistance in patients with type 2 diabetes leads to the accumulation of metabolites that can nonspecifically activate macrophages [60,61]. Diabetes is associated with impaired glucose metabolism, and hyperglycemic conditions lead to the accumulation of glycogen and an increase the apoptosis of β-cells [62]. Adipose tissue macrophages in obesity and type 2 diabetes increase TNF-α and IL-6 production in the events of a high amount of lipolysis [60,63]. In developing countries like India, T2DM is due to high intake of carbohydrate diet rather than high fat diet. To mimic non-obese T2DM, we developed chemically induced T2DM model using C57BL/6 mice and demonstrated excess inflammation in T2DM mice infected with *Mtb* leads to enhanced mortality [4].

*Mtb* infection reprograms macrophage metabolism by decelerating glycolysis and the TCA cycle [15]. Activation of macrophages by external stimulation leads to metabolic changes that increase glycolysis and reduce oxidative phosphorylation [64]. Diabetes is an immunometabolic disease, suggesting that TB-diabetes comorbidity further decompensates host cell immunometabolism resulting in accumulation of host toxic metabolites [34,65–68]. In the current study, metabolomic analysis indicated that 2-ketohexanoic acid was significantly more abundant in T2DM mouse alveolar macrophages infected with *Mtb* than in non-T2DM mouse alveolar macrophages infected with *Mtb* (Fig 3B–3C). In contrast, pyridoxine (vitamin B6)/4-pyridoxic acid was significantly less abundant in T2DM mouse alveolar macrophages infected with *Mtb* (Fig 3B–3C). 2-ketohexonic acid (2KH) significantly enhanced the expression of molecules involved in inflammation (both in vitro and in vivo), but marginally enhanced bacterial burden in the lungs of non-T2DM mice. Pyridoxine treatment unable to reduce bacterial burden (Fig 6F) and excess lung inflammation in T2DM mice infected with *Mtb* (Figs 6G and S6B–S6C). But Pyridoxine enhanced cas3 and cas8 expression of *Mtb*-infected T2DM alveolar macrophages (Fig 5D) and reduced RIPK3 and MLKL expression in the lungs of *Mtb*-infected T2DM mice (Fig 6H). In previous studies, we found mortality of *Mtb*-infected T2DM is due to excess inflammation rather than increased bacterial burden [4]. Our results suggest factors including host cell metabolism, immune response and plasticity to infection can contribute to initiation of inflammation in T2DM mice and 2KH and pyridoxine are two of these factors which may not be sufficient to impact bacterial burden in *Mtb*-infected mice [69–71].

Vitamin B6 deficiency is associated with several diseases, including diabetes, and the underlying mechanism is still under investigation [72]. Anti-TB drug, isoniazid (INH) depletes pyridoxine (vitamin B6) and causes the peripheral neuropathy [73,74]. In TB, supplementation with pyridoxine (vitamin B6) during isoniazid therapy is necessary in lactating women, individuals with HIV, alcoholism, malnutrition, diabetes mellitus patients with peripheral neuropathy and other diseases [74–77]. °Pyridoxal 5’ phosphate (known as active form of vitamin B6) deficiency in type 2 diabetic Drosophila models, causes severe chromosome and DNA damage and increases risk factor for developing cancer [78,79]. Vitamin B6 is known to prevent IL-1β secretion by inhibiting NLRP3 inflammatory activation and protects mice exposed to lethal endotoxic shock [80]. Even though pyridoxine had no effect on bacterial burden, our findings demonstrate that reduced pyridoxine/4-pyridoxic acid levels in *Mtb*-infected T2DM mice can contribute to necroptosis mediated inflammation (Figs 3, 5 and 6).

In conclusion, metabolic changes in T2DM mice lead to enhanced expression of TNFR1-mediated necroptosis and excess inflammation during *Mtb* infection. Further understanding of these molecular mechanism(s) provides important information to treat T2DM patients with latent tuberculosis infection or active TB disease.

## Materials and methods

### Ethics statement

All animal studies were approved by the Institutional Animal Care and Use Committee (IACUC) of the University of Texas Health Science Center at Tyler (protocol no. 645 and 717). The animal procedures involving the care and use of mice were undertaken in accordance with the guidelines of the NIH/OLAW (Office of Laboratory Animal Welfare).

### Animals

Specific pathogen-free 6-week-old female C57BL/6 mice were purchased from the Jackson Laboratory and housed at the animal facility at the University Texas Health Science Center at Tyler. All mice were maintained on a standard rodent chow diet (LabDiet, catalog 5053, 4.07 kcal/gm) during the entire experiment, and mice were housed randomly at 5 animals per cage in high-efficiency particulate air (HEPA)-filtered racks in certified animal biosafety level 2 (ABSL-2) and animal biosafety level 3 (ABSL-3) laboratories.

### Induction of T2DM

T2DM was induced by the combined administration of STZ and NA as described previously [4]. STZ was dissolved in 50 mM citric acid buffer and administered (180 mg/kg of body weight) intraperitoneally (i.p.) 3 times, with an interval of 10 days between doses. NA was dissolved in saline and administered i.p. (60 mg/kg of body weight) 15 minutes before the STZ injections. Mice were considered T2DM if their blood glucose level was ≥ 250 mg/dL, and control mouse blood glucose levels were regularly measured between 80 and 125 mg/dL.

### Antibodies and other reagents

For flow cytometry, we used anti-CD45, anti-CD11b, anti-MHCII, anti-CD11c, anti-F4/80, anti-TNFR1, anti-B220, anti-CD3, anti-CD4, anti-CD8 and anti-NK1.1 antibodies (all from BioLegend). For Western blotting, anti-MLKL (1:1000), anti-cleaved caspase 3 (1:1000), anti-cleaved caspase 8 (1:1000), anti-TNFRI (1:1000), β-actin (1:1000) and HRP conjugated secondary antibody (1:5000) were obtained from Cell Signaling Technology (Danvers, MA, USA). Anti-pMLKL (1:1000) was obtained from Sigma Aldrich (St. Louis, MO, USA). For confocal microscopy, we used anti-F4/80 (Abcam, Cambridge, UK), anti-pMLKL (Sigma Aldrich), anti-cleaved caspase 3 and anti-TNFR1 (Cell Signaling Technologies). Secondary antibodies (goat antirat IgG [H+L], Alexa 647 [catalog A21247]; goat antirabbit IgG [H+L], Alexa Fluor 488 [catalog A11008]; and goat anti-mouse IgG [H+L], Alexa Fluor 594 [catalog A11032]) were obtained from Invitrogen (Waltham, MA, USA), and Fluoroshield mounting medium with DAPI (Abcam, catalog ab104139) was used to stain nuclei. For neutralization studies, an anti-TNFR1 antibody was obtained from Thermo Fisher Scientific (Waltham, MA, USA). Streptozotocin (STZ), Nicotinamide (NA), deoxyadenosine monophosphate, acetyl choline, zVAD-FMK, Nec-1 and shikonin were obtained from Millipore Sigma (St. Louis, MO, USA). Pyridoxine and 2-Ketohexanoic acid were purchased from Cayman Chemicals.

### Aerosol infection with *Mtb* H37Rv and treatment with metabolites

For infection studies, control and T2DM mice were infected with *Mtb* H37Rv using an aerosol exposure chamber as described previously and determined bacterial burden [81,82]. For metabolite treatments, control mice uninfected or infected with *Mtb* received 2-ketohexanoic acid (20 mg/kg), and T2DM uninfected and T2DM infected with *Mtb* received pyridoxine (20 mg/kg) intranasally beginning at the day of infection. Treatment was continued every other day for 3 weekly doses for 1 week and 4 weeks and all the mice were euthanized at 1 week and 4 weeks post infection. Treatment dose for pyridoxine was determined based on previous studies [80] and we followed the same dose for 2-ketohexanoic acid and no adverse events found in mice.

### Culturing of alveolar macrophages and *Mtb* infection

Control and T2DM mice alveolar macrophages were isolated by bronchoalveolar lavage collection as described previously [4]. The adhered alveolar macrophages were infected with either *Mtb* H37Rv or GFP tagged *Mtb* H37Rv at a MOI of 1:2.5. For mRNA expression studies, the uninfected and infected macrophages were collected at 24h post infection. For protein studies, macrophages cell lysate was used at 72h post infection. For TNFR1 neutralization experiments, 10 μg/ml of anti-TNFR1 and IgG antibody was added to the cultures after 2h of *Mtb* infection and performed mRNA expression assays. 10 μM of pan-caspase inhibitor- zVAD-FMK, necroptosis inhibitor-Nec-1 and positive cell death control shikonin were used to rule out the cellular death mechanism in the alveolar macrophages with or without *Mtb* infection. For in vitro metabolite studies, the isolated alveolar macrophages were either treated with 50 μM of 2-ketohexanoic acid or pyridoxine.

### Preparation of lung cells and flow cytometry staining

Lungs were harvested from the PBS control and T2DM mice that were uninfected and *Mtb* H37Rv infected at the indicated time points and were placed into 60-mm dishes containing 2 mL of PBS (Thermo Fisher Scientific). The tissues were minced with scissors into pieces no larger than 2–3 mm, and the fluid was discharged onto a 40-μm filter that had been prewetted with 1 mL of PBS containing 0.5% BSA (Millipore Sigma) and suspended in a 50-mL conical tube (Thermo Fisher Scientific, 06-443-18). The syringe plunger was then used to gently disrupt the lung tissues before washing the filter with 2 mL of cold PBS/0.5% bovine serum albumin (BSA). For flow cytometry experiments, we gated on total leukocytes and measured various cell populations. For surface staining, ~1 × 10^6^ cells were resuspended in 200 μL of staining buffer and antibodies. The cells were then incubated at 4°C for 30 minutes with appropriate surface staining markers, washed twice, and fixed in 1% paraformaldehyde (Millipore Sigma) before acquisition using an Attune NxT acoustic flow cytometer (Invitrogen).

### Western blot

Cultured control and T2DM alveolar macrophages (uninfected and *Mtb*-infected) protein lysates were collected using MPER solution (Thermo; 78501) with 1x Halt Protease and phosphatase cocktail (Thermo; 78442). Protein concentrations was determined using Pierce BCA protein assay kit (Thermo; 23227). The samples were subjected to SDS-PAGE for separation and subsequently electroblotted onto PVDF membrane. After blocking with blocking buffer (Bio-Rad #12010020), the membrane was incubated with respective primary antibodies at 4°C overnight and followed by secondary antibody at 1h room temperature. Enhanced chemiluminescence detection method was used to visualize the protein bands.

### ELISA and LDH

TNF-a and IL-6 levels were measured in the culture supernatants and in the lung homogenates by enzyme-linked immunosorbent assay (ELISA) according to the manufacturer’s instructions. For multiplex ELISA determination, ProcartaPlex multiplex Immunoassay kit was used according to manufacturer instructions (ThermoFisher #EPX360-26092-901). LDH was measured in the culture supernatants using CyQUANT LDH Cytotoxicity kit (Thermo; C20301).

### Liquid chromatography–mass spectrometry (LC-MS)

Control and T2DM mice alveolar macrophages were isolated and infected with *Mtb* H37Rv at 1:2.5 ratio. After 72h, cell lysates were analyzed through LC-MS (UT Southwestern metabolomics core facility). Principal component analysis (PCA) and partial least squares discriminant analysis (PLS-DA) score plots were used to compare the metabolites from cultured alveolar macrophages of control, control *Mtb*, T2DM and T2DM *Mtb*. PCA, PLS-DA, Heat map and enrichment analysis was performed using MetaboAnalyst platform.

### Real-time PCR

Total RNA was extracted from mouse cultured alveolar macrophages or total lung using TRIzol (Invitrogen) according to the manufacturer’s instructions. RNA was reverse transcribed (iScript Reverse Transcription SuperMix for qPCR), and real-time PCR was performed using iTaq Universal SYBR Green Supermix (Bio-Rad, Hercules, CA, USA) according to the manufacturer’s instructions. Gene expression analysis was performed in a Bio-Rad CFX384 well system. All gene expression levels were normalized to β-actin/glyceraldehyde-3-phosphate dehydrogenase (GAPDH) internal controls in each sample, and the fold changes were calculated using the 2^-ΔΔCt^ method. The primers used in this study are listed in S1 Table.

### Histology

At the specified time points, mice were euthanized, and the harvested lungs were inflated and fixed in 10% neutral buffered formalin for 48 hours to inactivate the infectious agents. Paraffin-embedded blocks were cut into 5-μm-thick sections and hematoxylin and eosin (H&E) staining was performed. Lung lesions were quantified by calculating percentage of lesion areas per microscopic field using NIH ImageJ software and the values were normalized with either PBS or T2DM controls.

### Confocal microscopy

Confocal microscopy was performed to determine the expression of apoptotic (cleaved caspase 3) or necroptotic (pMLKL) expressing macrophages (F4/80+). The lung tissues were stored in 10% neutral buffered formalin; then, the samples were paraffin embedded and cut into 5-μm-thick sections that were deparaffinized and rehydrated. The tissue sections were subjected to heat-induced antigen retrieval in 10 mM sodium citrate buffer (pH 6.0). Then, the lung tissue sections were incubated in 0.025% Triton X-100 in PBS with Tween 20 (PBST) for 10 minutes and washed 3 times for 5 minutes each (3 × 5 minutes) using PBS. Nonspecific binding was blocked with 5% goat serum or BSA in PBST for 1 hour, and the slides were washed 2 × 5 minutes with PBST. The slides were then incubated at 4°C overnight with the appropriate dilutions (diluted in 1% BSA/1x PBST) of the following primary antibodies: anti-F4/80 (1:200), anti-cleaved caspase 3 (1:200) and anti-pMLKL (1:200); subsequently, the slides were washed thoroughly 3 × 5 minutes with PBST. Then, the tissue sections were stained with the respective secondary antibodies at 1:1000 dilutions (v/v), washed again with PBST for 3 × 5 minutes, and mounted with fluoroshield mounting medium with DAPI. The slides were then examined and analyzed under a laser scanning confocal microscope (Zeiss LSM 510 Meta). An IgG isotype secondary control was used for all confocal microscopy studies, and Zen 2009 software (Carl Zeiss) was used for image acquisition. The images were processed uniformly for each experiment using ImageJ National Institutes of Health (NIH) software. Representative images from *n* = 5 mice/group are shown.

### Statistics

Data analyses were performed using GraphPad Prism 9.0 (GraphPad Software Inc.). The results are expressed as the mean ± standard deviation (SD). The statistical analysis was performed by one-way ANOVA followed by Tukey’s multiple comparison test or unpaired two tailed t-test. *p* < 0.05 was considered significant.

## Supporting information

S1 Fig mRNA expression of cell death pathways in *Mtb* H37Rv-infected control and T2DM mouse alveolar macrophages. Alveolar macrophages (AMs) from control and T2DM mice were isolated and infected with *Mtb* H37Rv as described in the methods section. After 24 h of postinfection, the gene expression of Caspase 3, Caspase 8, RIPK3, MLKL, Atg7/5, Caspase 11 and Gpx4 was determined by qRT-PCR. Three independent experiments were performed. Each independent experiment was performed using pooled AMs from 3 to 5 mice in each group. The data are shown as the mean ± standard deviation (SD). The statistical analysis was performed by one-way ANOVA followed by Tukey’s multiple comparison test. ***, p<0.001 and ****, p<0.0001.
(TIF)

S2 Fig Metabolic profile and pathway analysis of *Mtb* H37Rv-infected control and T2DM mouse alveolar macrophages. AMs from control and T2DM mice were isolated and infected with *Mtb* H37Rv. After 72 h, cell lysates were analyzed using LC/MS (A) Heatmap shows the total metabolites screened. (B) Pathway enrichment analysis was performed using MetaboAnalyst 4.0.
(TIF)

S3 Fig Assessment of metabolite cytotoxicity and TNFR1 expression in *Mtb*-infected alveolar macrophages. Control mouse AMs were treated with different concentrations of 2-ketohexanoic acid (2KH), pyridoxine (PX), deoxyadenosine monophosphate (dAMP) and acetylcholine (Ach). (A) After 72 h, the survival percentage was determined by LDH release. (B) TNFR1 expression was determined by qRT-PCR in *Mtb*-infected control mice (dAMP at 50 μM concentration) and T2DM mice (Ach at 50 μM concentration) alveolar macrophages. Each independent experiment was performed using pooled AMs from 3 to 5 mice in each group. The data are shown as the mean ± standard deviation (SD).
(TIF)

S4 Fig Metabolites treatment alters cytokines/chemokines and myeloid cell profile in the lungs of *Mtb*-infected mice at 1-week post infection. As mentioned in the methods sections, some of the *Mtb*-infected T2DM mice were treated with 2-ketohexanoic acid (20 mg/kg of body weight) or pyridoxine (20 mg/kg of body weight) intranasally. One-week postinfection (A-B) Bacterial burden in the lungs was measured (C) Bar graphs represent the various immune cell populations (Myeloid cells, T cells, NK and B cells). Cell numbers were normalized by 10^6^ cells in the lungs. The data are shown as the mean ± standard deviation (SD). The statistical analysis was performed by one-way ANOVA followed by Tukey’s multiple comparison test. *, p<0.05 and **, p<0.01. (D) lung homogenates were collected, and cytokines/chemokines were estimated using 36-plex ELISA kit by Luminex and the heatmap data is presented. n = 3 mice per group were used.
(TIF)

S5 Fig In vivo treatment with metabolites alters apoptosis and TNFR1 expression of *Mtb*-infected mouse lung cells. As mentioned in the methods sections, some of the *Mtb*-infected T2DM mice were treated with 2-ketohexanoic acid (20 mg/kg of body weight) or pyridoxine (20 mg/kg of body weight) intranasally. One-month postinfection (A) Expression of Cas3 and Cas8 (apoptotic) was determined in the lungs by qRT-PCR. Five mice per group were used for each independent experiment. The data are shown as the mean ± standard deviation (SD). The statistical analysis was performed by one-way ANOVA followed by Tukey’s multiple comparison test. **, p<0.01 and ***, p<0.001. (B) A representative flow cytometry gating strategy is shown for alveolar macrophages expressing TNFR1 in the lungs of *Mtb*-infected mice.
(TIF)

S6 Fig In vivo treatment with 2KH and pyridoxine influences lungmyeloid cells and inflammation in *Mtb*-infected mice at 1-month post infection. As mentioned in the methods sections, some of the *Mtb*-infected T2DM mice were treated with 2-ketohexanoic acid (20 mg/kg of body weight) or pyridoxine (20 mg/kg of body weight) intranasally. (A) Bar graphs represent the myeloid cell populations (CD11b+, CD11c+, CD11b+CD11c+ and CD11c+F4/80+ cells). Cell numbers were normalized by 10^6^ cells in the lungs. (B) One-month postinfection, paraffin-embedded tissue sections were prepared, and hematoxylin and eosin staining was performed. (C) Lung lesions were quantified by calculating percentage of lesion areas per microscopic field and values were normalized with either PBS or T2DM controls and bar graphs were shown. Data are representative of two independent experiments. Five mice per group were used for each independent experiment. The data are shown as the mean ± standard deviation (SD). The statistical analysis was performed by one-way ANOVA followed by Tukey’s multiple comparison test. **, p<0.01 and ****, p<0.0001.
(TIF)

S1 Table List of qRT-PCR primers used in this study. (DOC)
