# Supplementary figures and images for: Metabolic changes enhance necroptosis of type 2 diabetes mellitus mice infected with Mycobacterium tuberculosis
Source: PLoS Pathog. 2024 May 10;20(5):e1012148. doi: 10.1371/journal.ppat.1012148 (PMC11086854; doi:10.1371/journal.ppat.1012148)

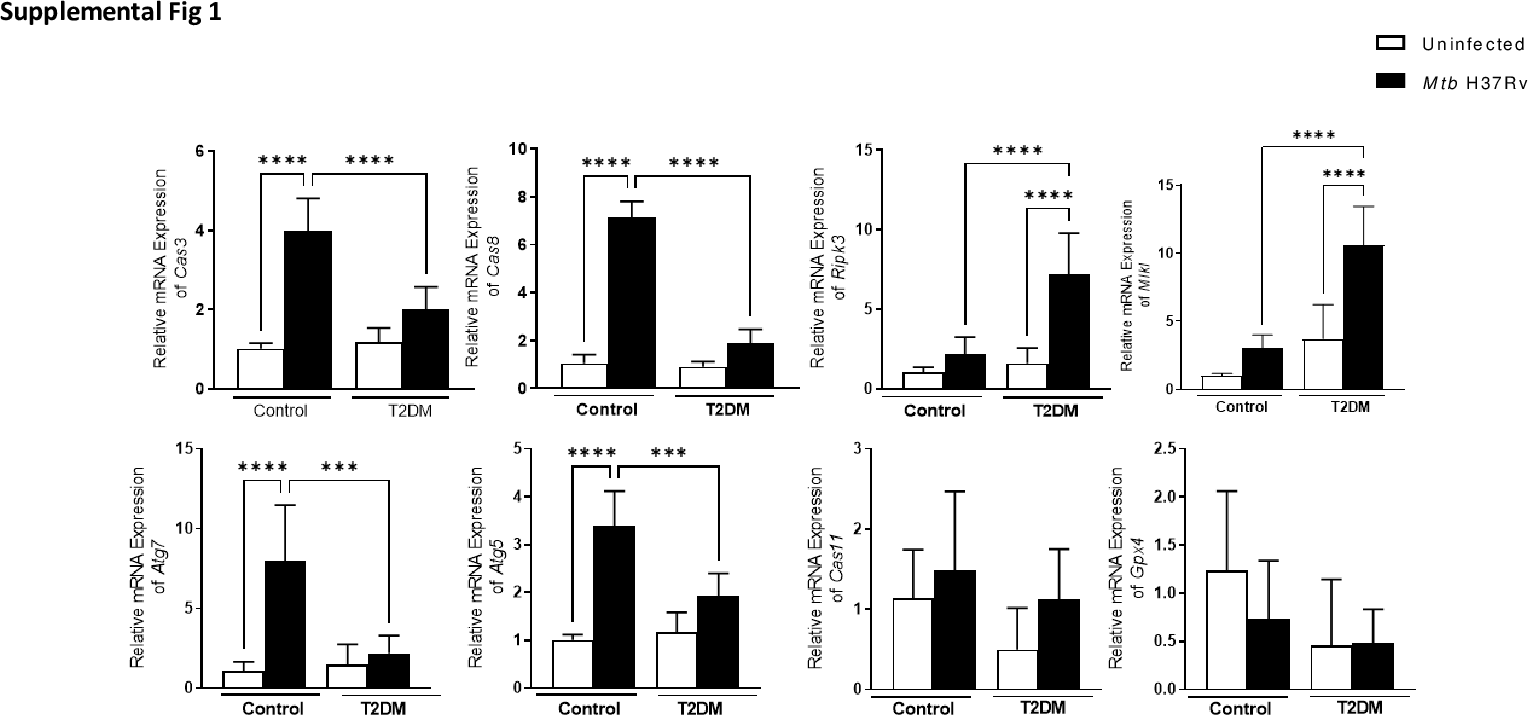

Supplement: S1 Fig — Alveolar macrophages (AMs) from control and T2DM mice were isolated and infected with Mtb H37Rv as described in the methods section. After 24 h of postinfection, the gene expression of Caspase 3, Caspase 8, RIPK3, MLKL, Atg7/5, Caspase 11 and Gpx4 was determined by qRT-PCR. Three independent experiments were performed. Each independent experiment was performed using pooled AMs from 3 to 5 mice in each group. The data are shown as the mean ± standard deviation (SD). The statistical analysis was performed by one-way ANOVA followed by Tukey’s multiple comparison test. ***, p<0.001 and ****, p<0.0001. (TIF) [file ppat.1012148.s001.tif]

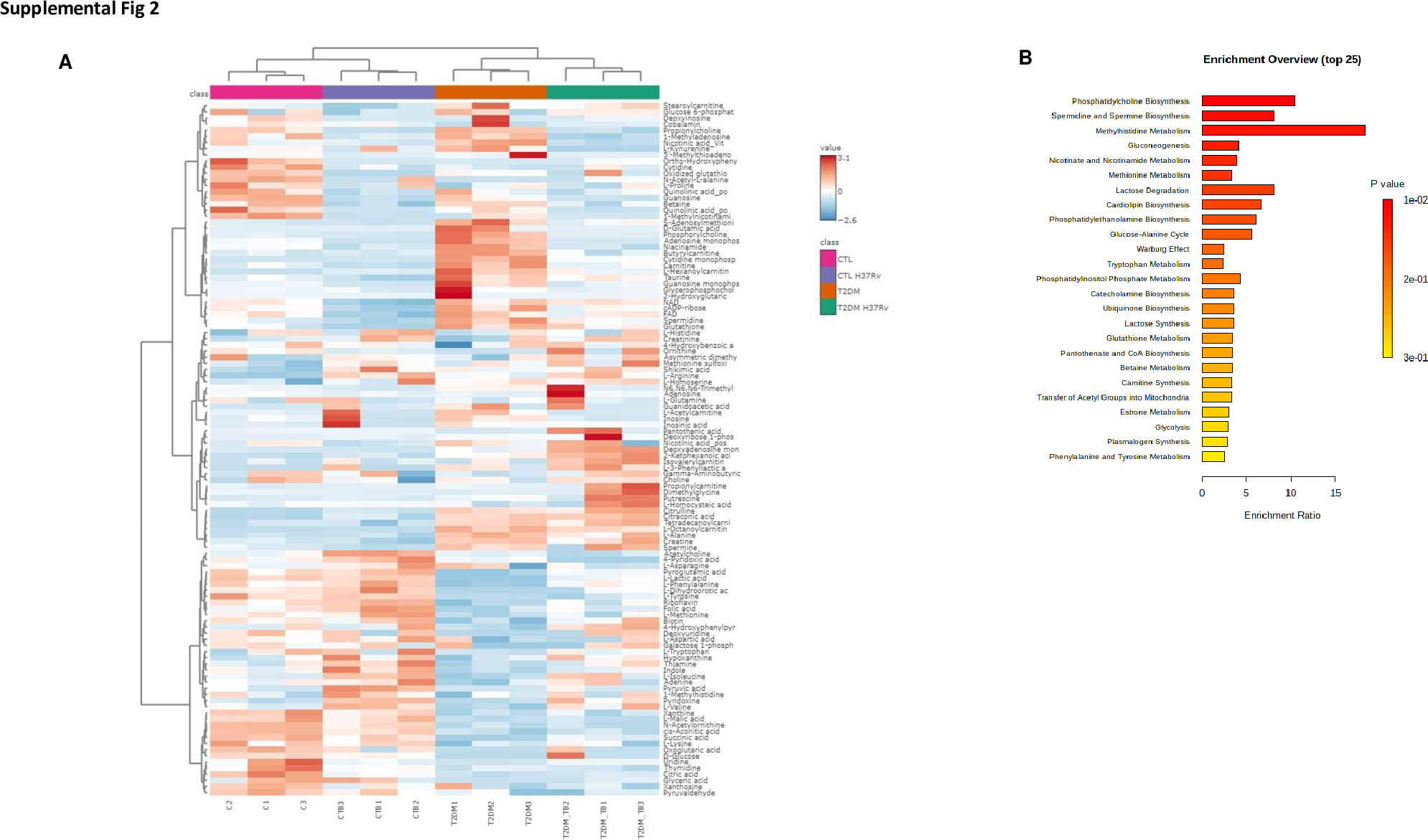

Supplement: S2 Fig — AMs from control and T2DM mice were isolated and infected with Mtb H37Rv. After 72 h, cell lysates were analyzed using LC/MS (A) Heatmap shows the total metabolites screened. (B) Pathway enrichment analysis was performed using MetaboAnalyst 4.0. (TIF) [file ppat.1012148.s002.tif]

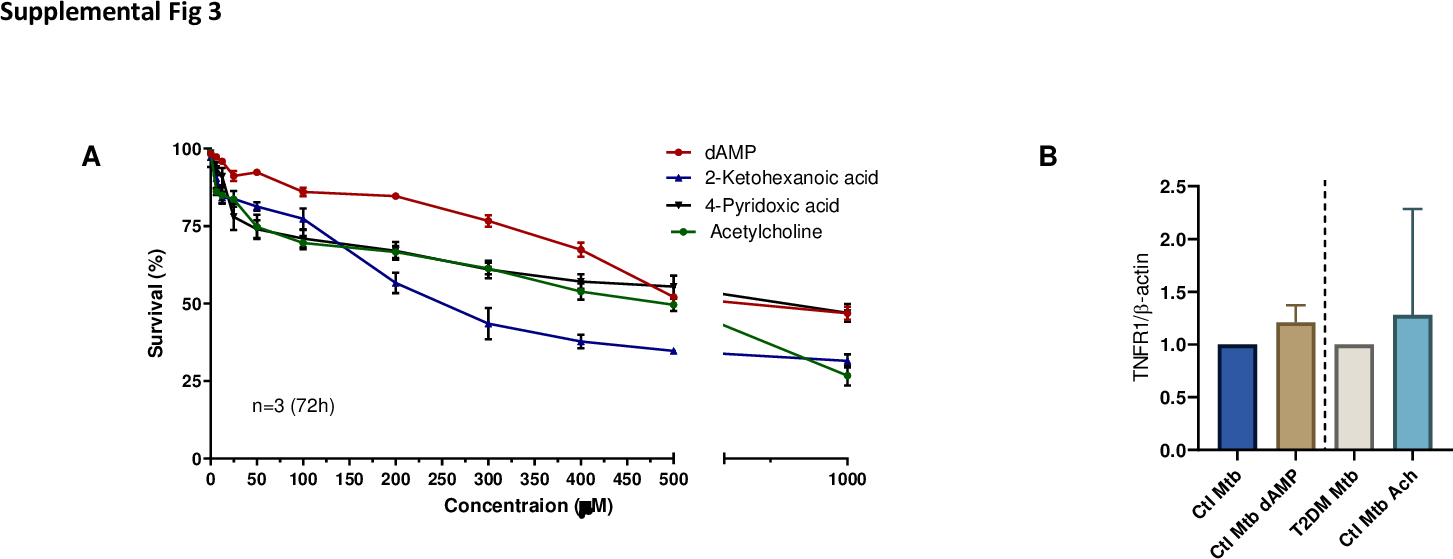

Supplement: S3 Fig — Control mouse AMs were treated with different concentrations of 2-ketohexanoic acid (2KH), pyridoxine (PX), deoxyadenosine monophosphate (dAMP) and acetylcholine (Ach). (A) After 72 h, the survival percentage was determined by LDH release. (B) TNFR1 expression was determined by qRT-PCR in Mtb-infected control mice (dAMP at 50 μM concentration) and T2DM mice (Ach at 50 μM concentration) alveolar macrophages. Each independent experiment was performed using pooled AMs from 3 to 5 mice in each group. The data are shown as the mean ± standard deviation (SD). (TIF) [file ppat.1012148.s003.tif]

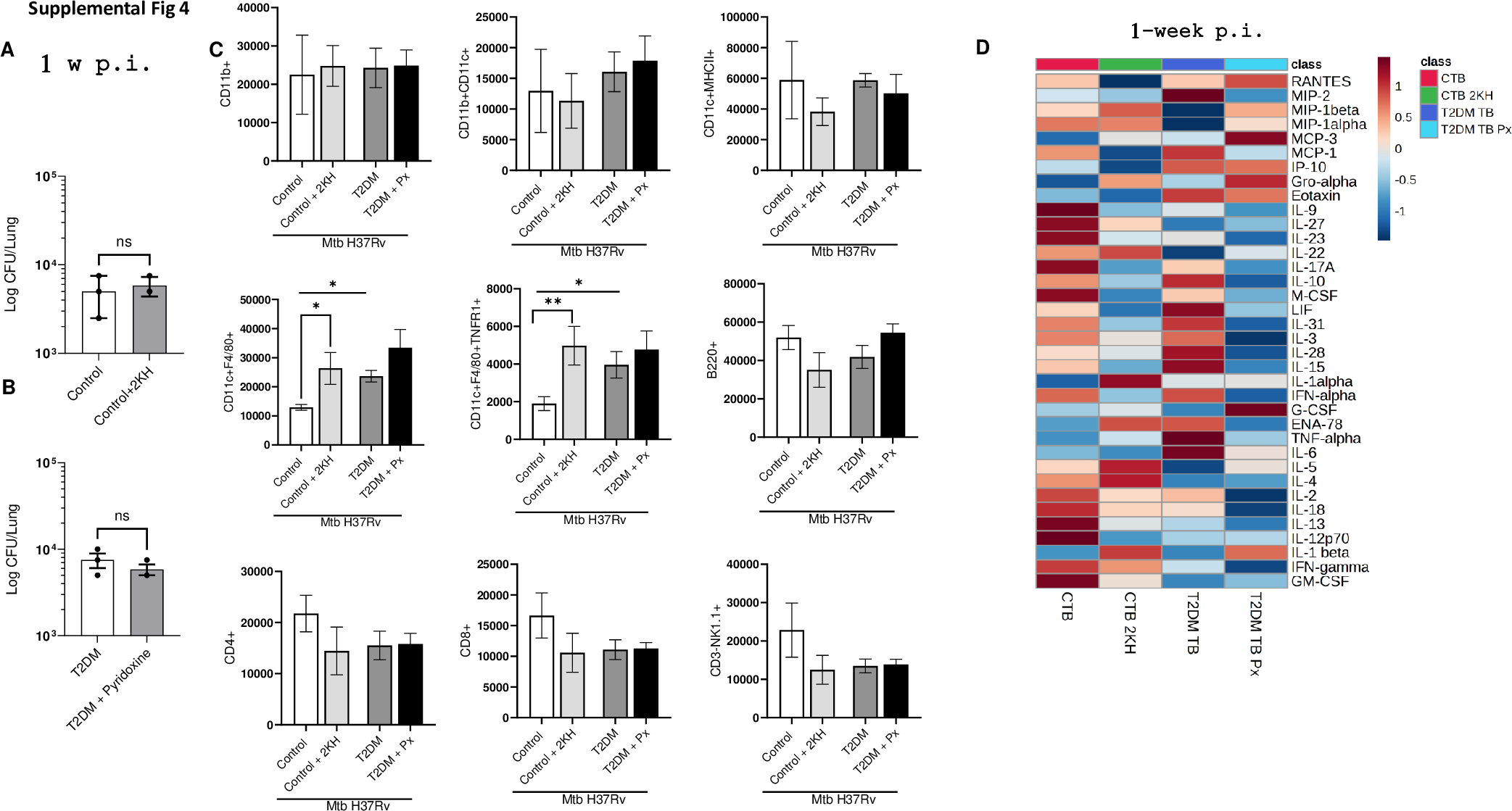

Supplement: S4 Fig — As mentioned in the methods sections, some of the Mtb-infected T2DM mice were treated with 2-ketohexanoic acid (20 mg/kg of body weight) or pyridoxine (20 mg/kg of body weight) intranasally. One-week postinfection (A-B) Bacterial burden in the lungs was measured (C) Bar graphs represent the various immune cell populations (Myeloid cells, T cells, NK and B cells). Cell numbers were normalized by 106 cells in the lungs. The data are shown as the mean ± standard deviation (SD). The statistical analysis was performed by one-way ANOVA followed by Tukey’s multiple comparison test. *, p<0.05 and **, p<0.01. (D) lung homogenates were collected, and cytokines/chemokines were estimated using 36-plex ELISA kit by Luminex and the heatmap data is presented. n = 3 mice per group were used. (TIF) [file ppat.1012148.s004.tif]

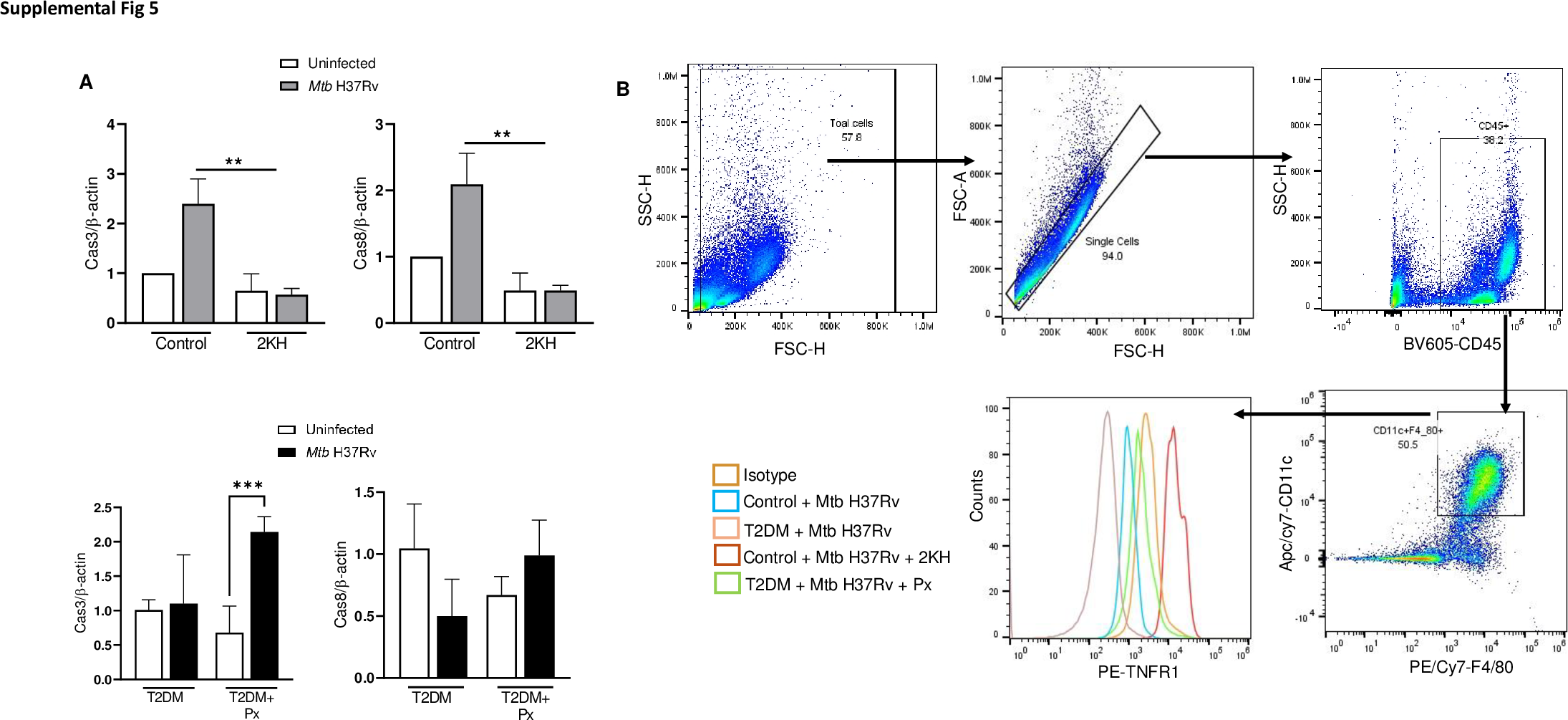

Supplement: S5 Fig — As mentioned in the methods sections, some of the Mtb-infected T2DM mice were treated with 2-ketohexanoic acid (20 mg/kg of body weight) or pyridoxine (20 mg/kg of body weight) intranasally. One-month postinfection (A) Expression of Cas3 and Cas8 (apoptotic) was determined in the lungs by qRT-PCR. Five mice per group were used for each independent experiment. The data are shown as the mean ± standard deviation (SD). The statistical analysis was performed by one-way ANOVA followed by Tukey’s multiple comparison test. **, p<0.01 and ***, p<0.001. (B) A representative flow cytometry gating strategy is shown for alveolar macrophages expressing TNFR1 in the lungs of Mtb-infected mice. (TIF) [file ppat.1012148.s005.tif]

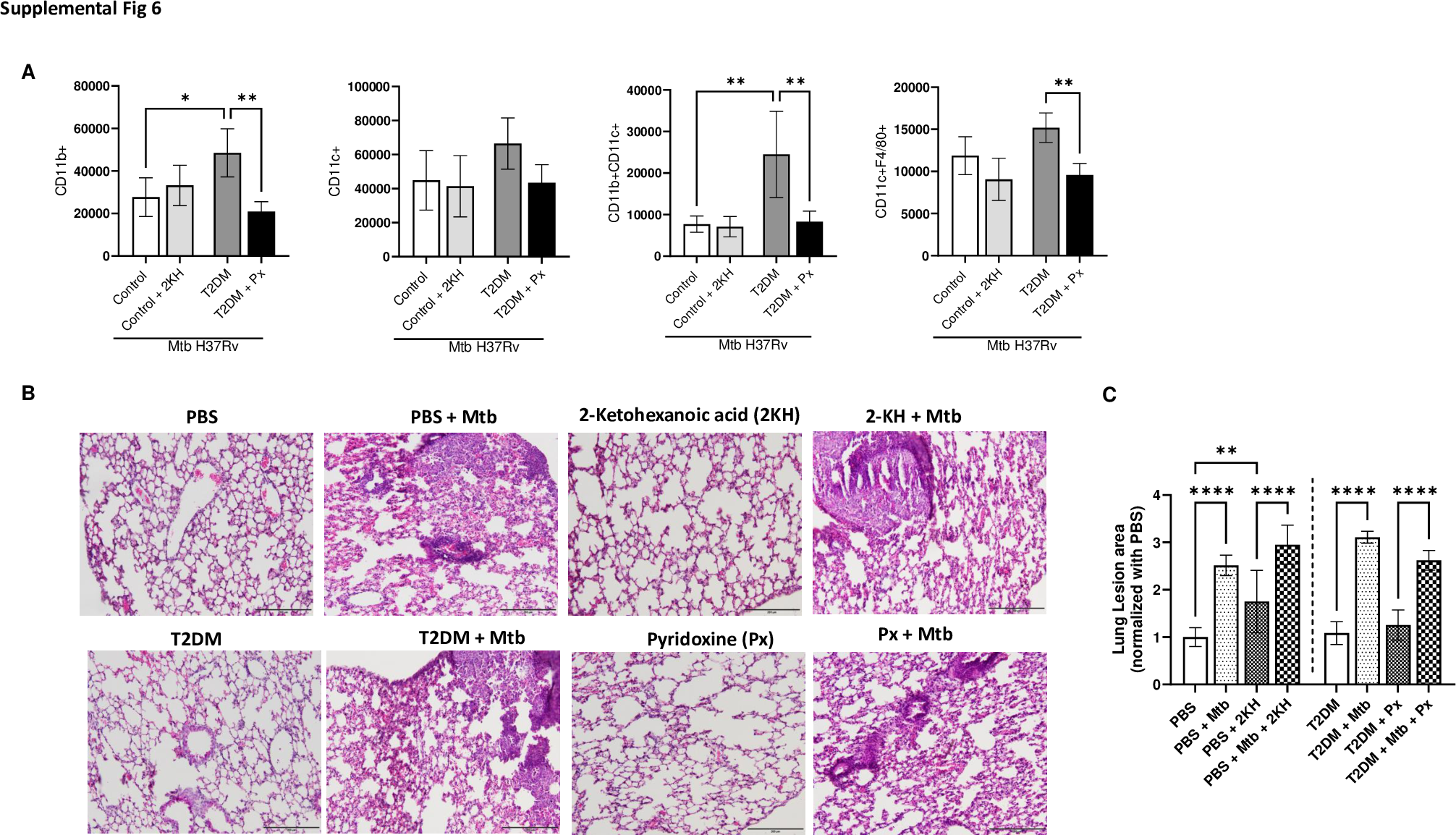

Supplement: S6 Fig — As mentioned in the methods sections, some of the Mtb-infected T2DM mice were treated with 2-ketohexanoic acid (20 mg/kg of body weight) or pyridoxine (20 mg/kg of body weight) intranasally. (A) Bar graphs represent the myeloid cell populations (CD11b+, CD11c+, CD11b+CD11c+ and CD11c+F4/80+ cells). Cell numbers were normalized by 106 cells in the lungs. (B) One-month postinfection, paraffin-embedded tissue sections were prepared, and hematoxylin and eosin staining was performed. (C) Lung lesions were quantified by calculating percentage of lesion areas per microscopic field and values were normalized with either PBS or T2DM controls and bar graphs were shown. Data are representative of two independent experiments. Five mice per group were used for each independent experiment. The data are shown as the mean ± standard deviation (SD). The statistical analysis was performed by one-way ANOVA followed by Tukey’s multiple comparison test. **, p<0.01 and ****, p<0.0001. (TIF) [file ppat.1012148.s006.tif]
