# Supplementary material for: Metabolic changes enhance necroptosis of type 2 diabetes mellitus mice infected with Mycobacterium tuberculosis
Source: PLoS Pathog. 2024 May 10;20(5):e1012148. doi: 10.1371/journal.ppat.1012148 (PMC11086854; doi:10.1371/journal.ppat.1012148)
Supplement: S1 Table — (DOC) [file ppat.1012148.s007.doc]

**Supplemental Table 1.**

| Gene | Forward Primer | Reverse Primer |
| --- | --- | --- |
| TLR4 | CCCATGCATTTGGCCTTAGC | AGAGCACTGAACCTCCTTGC |
| Gapdh | GATGACATCAAGAAGGTGGTGA | TGCTGTAGCCCGTATTCATTGTC |
| βactin | TTACAGGAAGTCCCTCACCC | ACACAGAAGCAATGCTGTCAC |
| TNFR1 | GGGCACCTTTACGGCTTCC | GGTTCTCCTTACAGCCACACA |
| Ripk3 | CACATACTTTACCCTTCAGA | TCAGAACAGTTGTTGAAGAC |
| Ripk1 | CGTGAGAATATTAAGAGTGC | TGTACCTGTAGTTCCAAATC |
| IL-6 | CACAAAGCCAGAGTCCTTCAGAGA | CTAGGTTTGCCGAGTAGATCT |
| TNFα | CCTGTAGCCCACGTCGTAGC | AGCAATGACTCCAAAGTAGACC |
| Casp8 | CTAGACTGCAACCGAGAGG | GCAGGCTCAAGTCATCTTCC |
| Casp3 | GAGCTGGACTGTGGCATTGA | CCACCGGTATCTTCTGGCAA |
| Mlkl | GACCAAACTGAAGACAAGTA | CTCACTATTCCAACACTTTC |
